# The Effect of Hearing Loss and Hearing Device Fitting on Fatigue in Adults: A Systematic Review

**DOI:** 10.1097/AUD.0000000000000909

**Published:** 2020-07-02

**Authors:** Jack A. Holman, Avril Drummond, Graham Naylor

**Affiliations:** 1 Hearing Sciences (Scottish Section), Division of Clinical Neuroscience, School of Medicine, University of Nottingham, Glasgow, United Kingdom; 2 School of Health Sciences, University of Nottingham, Nottingham, United Kingdom.

**Keywords:** Cochlear implants, Fatigue, Hearing aids, Hearing devices, Hearing impairment, Hearing loss, Systematic review

## Abstract

To conduct a systematic review to address two research questions: (Q1) Does hearing loss have an effect on fatigue? (Q2) Does hearing device fitting have an effect on fatigue? It was hypothesized that hearing loss would increase fatigue (H1), and hearing device fitting would reduce fatigue (H2).

**Design:**

Systematic searches were undertaken of five bibliographic databases: Embase, MedLine, Web of Science, Psychinfo, and the Cochrane Library. English language peer-reviewed research articles were included from inception until present. Inclusion and exclusion criteria were formulated using the Population, Intervention, Comparison, Outcomes and Study design strategy.

**Results:**

Initial searches for both research questions produced 1,227 unique articles, after removal of duplicates. After screening, the full text of 61 studies was checked, resulting in 12 articles with content relevant to the research questions. The reference lists of these studies were examined, and a final updated search was conducted on October 16, 2019. This resulted in a final total of 20 studies being selected for the review. For each study, the information relating to the Population, Intervention, Comparison, Outcomes and Study design criteria and the statistical outcomes relating to both questions (Q1 and Q2) were extracted. Evidence relating to Q1 was provided by 15 studies, reporting 24 findings. Evidence relating to Q2 was provided by six studies, reporting eight findings. One study provided evidence for both. Using the Grading of Recommendations Assessment, Development and Evaluation guidelines, the quality of evidence on both research questions was deemed to be “very low.” It was impossible to perform a meta-analysis of the results due to a lack of homogeneity.

**Conclusions:**

As the studies were too heterogeneous to support a meta-analysis, it was not possible to provide statistically significant evidence to support the hypotheses that hearing loss results in increased fatigue (H1) or that hearing device fitting results in decreased fatigue (H2). Despite this, the comparative volume of positive results and the lack of any negative findings are promising for future research (particularly in respect of Q1). There was a very small number of studies deemed eligible for the review, and there was large variability between studies in terms of population, and quantification of hearing loss and fatigue. The review highlights the need for consistency when measuring fatigue, particularly when using self-report questionnaires, where the majority of the current evidence was generated.

## Abstract 2

Supplemental Digital Content is available in the text.

## INTRODUCTION

The detrimental effects of hearing loss go beyond reduced audibility, problems with speech recognition and subsequent communication difficulties. Evidence has shown that hearing loss is also related to poorer psychosocial outcomes. People with a hearing loss can experience impaired social interactions, which may lead to reduced participation and benefit from social situations (Chia et al. 2007; Ciorba et al. 2012). The reduction in communicative performance brought about by poorer speech recognition can lead to a requirement for increased listening effort (cognitive exertion) to maintain performance (Kramer et al. 1997; Desjardins & Doherty 2013). A growing body of literature has shown that some people with a hearing loss exhibit hearing loss-related fatigue (Hétu et al. 1988; Bess & Hornsby 2014; Holman et al. 2019). The extra listening effort experienced by people with a hearing loss has been linked to these increased levels of fatigue when compared to people who do not have a hearing loss (Hornsby & Kipp 2016; Alhanbali et al. 2017). It has been suggested that the increased cognitive load, due to the extra effort necessary to process the speech signal, will deplete finite cognitive resources and result in fatigue (Kahneman 1973; Shinn-Cunningham & Best 2008). There is, however, no concrete proof of such a causal link in the literature (McGarrigle et al. 2014; Hornsby et al. 2016).

While hearing loss-related fatigue can be a major problem for some people, this is not universally the case for all those with hearing loss. Previous research has identified additional factors, relevant to hearing loss, which could potentially modulate fatigue. These factors include motivation, negative emotions, the varied lifestyles of people, and differing utilization of coping strategies to alleviate difficulty in challenging listening situations (Barnes & Van Dyne 2009; Herlambang et al. 2019; Holman et al. 2019). While there has been a recent increase in publications which support previous anecdotal evidence of a link between hearing loss and fatigue, the results are not as consistent as might have been expected.

One of the possible reasons that inconsistent results are found is that fatigue itself is not a simple construct. Fatigue can be viewed as transient (momentary and task related) or long term (not specifically task related; Hockey 2013). Motivation, for example, could have a direct influence on transient fatigue, whereas any effect on long-term fatigue would be much harder to ascertain. Additionally, it has been argued that fatigue is a multi-dimensional construct. Physical fatigue, mental fatigue, emotional fatigue, and vigor/vitality have been identified as separate dimensions of fatigue (Stein et al. 2004). However, whether people consistently respond to these separate dimensions of fatigue is debated (Michielsen et al. 2003).

Hearing loss-related fatigue can be assessed either subjectively via self-report questionnaires or objectively via physiological or behavioral methods (McGarrigle et al. 2014). While these methodologies superficially appear to be measuring the same thing, a lack of correlation between outcomes suggests that they may not be (Hornsby 2013). The most common methodology utilized to measure fatigue in people with a hearing loss is self-report questionnaires (Hornsby & Kipp 2016; Alhanbali et al. 2017, 2018; Wang et al. 2018). Studies investigating fatigue and hearing loss use varying terminologies and variables related to fatigue such as “need for recovery” (a measure of the deficit in energy and performance potential caused by fatigue after exertion; van Veldhoven & Broersen 2003) and “vitality” (the state of being active, synonymous with energy and measured in the opposite direction to fatigue; Ryan & Frederick 1997). Consequently, the conclusions drawn from such studies may be less directly attributable to fatigue. Very few studies have used behavioral and physiological methods to study hearing loss-related fatigue. Behavioral measures of fatigue investigate performance decrement over time, often using multiple task paradigms (Hornsby 2013). Physiological methods involve the use of markers such as pupillary responses to investigate listening effort and thus potentially fatigue. Measurement of listening effort and fatigue using physiological methods has been identified as possible in normal-hearing populations; however, the research is in its infancy (Hopstaken et al. 2015; McGarrigle et al. 2017b; Herlambang et al. 2019).

To alleviate some of the difficulty with speech perception that is synonymous with hearing loss, hearing devices are commonly fitted. These devices are most commonly hearing aids or cochlear implants. The fitting of hearing devices usually leads to large reductions in hearing handicap (Ferguson et al. 2017). Hearing aid and cochlear implant fitting have also been shown to reduce the listening effort required in conversational situations (Pals et al. 2013; Picou et al. 2013; Wu et al. 2014). Given the postulated links between listening effort and fatigue, it would be expected that hearing device fitting would result in reduced fatigue.

Given the diversity of conceptualizations, terminologies, and study types, it is important to establish what the existing knowledge base is so that future research into hearing loss-related fatigue can be designed in the most effective and efficient manner. The optimum way to accomplish this is using systematic review methodology to investigate (1) the impact of hearing loss on levels of fatigue and (2) whether hearing device fitting influences levels of fatigue. The objective of the current systematic review was to address two research questions:

Q1) Does hearing loss have an effect on fatigue?

Q2) Does hearing device fitting have an effect on fatigue?

It was hypothesized that hearing loss results in increased fatigue (H1) and that hearing device fitting results in reduced fatigue (H2).

## MATERIALS AND METHODS

The “preferred reporting items for systematic reviews and meta-analyses” (PRISMA) were utilized in this review to ensure full and transparent conduct and reporting of the systematic review (Moher et al. 2009).

### Search Strategy

Systematic searches were undertaken of the bibliographic databases of Embase, MedLine, Web of Science, Psychinfo, and the Cochrane Library. The search variables used included control terms and free-text terms. Two focused questions were the basis of the searches, namely: (Q1) Does hearing loss have an effect on fatigue? (Q2) Does hearing device fitting have an effect on fatigue? All peer-reviewed research articles identified were included initially from inception until February 1, 2017, with later searches including all recent articles until October 16, 2019. The search terms can be found in Supplemental Digital Content 1, http://links.lww.com/EANDH/A673.

### Inclusion and Exclusion

To determine the inclusion and exclusion criteria for the study, the Population, Intervention, Comparison, Outcomes and Study design (PICOS) strategy was utilized (Armstrong 1999). The use of the PICOS strategy to identify high-quality relevant evidence has been demonstrated by numerous studies. The PICOS criteria that were used to determine which studies to include in the review were as follows:

#### Population

Adults aged at least 18 years old with a hearing loss. This could be measured audiometrically or by other means such as self-reported hearing loss.

#### Intervention

The exposure variable of interest (“Intervention” in the PICOS framework) was hearing loss (Q1) or hearing device fitting (Q2). Hearing loss as an exposure variable was considered as either the presence of a hearing loss or the level of hearing loss. Measurement of hearing loss could be self-reported hearing difficulty, pure tone audiometric assessment, or indirect measures such as hearing handicap (i.e., difficulties caused by hearing loss). A broad range of measures of the presence of a hearing loss was provided to include studies that did not primarily investigate hearing loss and fatigue. Hearing device fitting was considered either as aided versus unaided or as one versus two hearing devices. This included, but was not limited to, hearing aids and cochlear implants.

#### Comparison

The comparison of interest in studies was a lack of, or varied levels of, the exposure variable (intervention). The possible alternatives for comparison with the intervention were between-group comparisons (control group with no hearing loss or no hearing device), within-group comparison (e.g., the effect of level of hearing loss), or within-subjects repeated measures designs (e.g., with and without, or before and after, hearing devices).

#### Outcomes

The primary outcome was the level of fatigue (acute or long term). This could be either directly through self-report questionnaires (subjective), through the reduction in performance over time on tasks (behavioral), or through changes in physical response (physiological). To avoid an unmanageably large and unproductive initial search result, these criteria were translated into practicable search terms by requiring that in order for a study to be included, it had to mention some form of fatigue terminology. Due to the use of widely differing fatigue terminology and lack of clear consensus on how to classify fatigue, indirect measures of subjective fatigue were included. Terms referring to a reduction in energy or vitality were included, for example, “lack of energy” or “need for recovery.” The full range of terms used by studies was not known prior to the searches.

#### Study Design

All studies were English language peer-reviewed research studies. Types of study to be included were randomized controlled trials (RCTs), non-RCTs, experimental studies with repeated measures design, and observational studies. Qualitative studies were not included.

The selection of studies went through several stages: First, all articles identified via the database searches were screened for duplicates, which were then removed. The updated list of studies was then screened for potential relevance through inspection of titles and abstracts by one author (J.H.). To ensure that no studies were rejected accidentally, the author and one other researcher (G.N.) then independently used the full texts of all remaining articles for categorization. Studies were categorized as “yes” when they were definitely relevant, “maybe” where there was some doubt about at least one criterion, and “no” when it was deemed that the study did not meet the criteria for inclusion and there was no need for further discussion. This technique was utilized by Knudsen et al. (2010) and Ohlenforst et al. (2017). The two independent categorization lists were then compared, and any differences were discussed and subsequently resolved. Nineteen studies were discussed in this manner and a decision was agreed upon for all, with 17 found not to meet the criteria for inclusion. Had no agreement been found at any point, a third reviewer would have supported the final decision on categorization; however, this was not necessary. Next, the reference lists of the relevant articles were examined to identify any additional relevant articles. Updated searches were conducted on March 1, 2019, and October 16, 2019, to include recent articles, which were scrutinized in the same manner as mentioned earlier. The systematic review protocol was uploaded to the PROSPERO international prospective register of systematic reviews (https://www.crd.york.ac.uk/PROSPERO, registration number: CRD42018090924).

### Data Extraction and Analysis

For all relevant articles, the information related to PICOS was extracted: population details, population numbers, method and criteria for measuring hearing loss or hearing device fitting, comparison population details, method of measuring fatigue, study type, and study outcomes. The outcome measures relating to fatigue were categorized as either subjective, behavioral, or physiological measures. The results were then coded based on their addressing of the hypotheses of Q1 or Q2. The hypothesis of Q1 stated that “hearing loss is associated with higher levels of fatigue.” This included both the presence of a hearing loss, as well as the severity of hearing loss. The hypothesis of Q2 stated that “hearing device fitting is associated with lower levels of fatigue.” Results from the selected studies that supported the hypothesis were listed as “supported” (+). Results that showed no statistical support were listed as “no effect” (=). Where results showed the opposite effect to the hypothesis, they were listed as “negative effect” (-). Each study could provide more than one result relating to Q1 or Q2.

It was not possible to conduct a meta-analysis across studies based on outcome measures, as the studies were too heterogeneous. This was true of most of the key PICOS criteria such as population demographics, measurement and classification of hearing loss, outcome measures used to measure fatigue, and study design. Despite this, it was possible to make comparisons between the studies based on the support or otherwise of the hypotheses, as well as the descriptive information extracted from studies in the form of PICOS information. The study findings and evidence quality were considered to give an informed interpretation of the studies.

### Evidence Quality

The rating of the quality of the evidence provided by the selected studies was derived from the Grading of Recommendations Assessment, Development and Evaluation (GRADE) guidelines (Guyatt et al. 2011b). The GRADE framework is a widely used tool for grading the quality of evidence, adopted by organizations such as the Cochrane Collaboration (Higgins & Green 2011). Rather than providing a measurement of the individual studies themselves, the quality of evidence is measured for each type of outcome measurement type across all studies. GRADE gives an overall score of the quality of the evidence. The possible levels of evidence quality are “very low,” “low,” “moderate,” and “high.” Evidence from RCTs starts at high quality, whereas any measures that include observational studies start at low quality. From these starting points, the quality of the evidence can be raised or lowered depending on the scoring of five criteria. The criteria by which the outcome measures are rated for quality are “risk of bias,” “inconsistency,” “indirectness,” “imprecision,” and “publication bias.” While diversity between studies in factors such as population type can be a good thing, with regards to the GRADE criteria inconsistency detracts from the generalizability of results. For each type of outcome measurement across all studies, a rating is given based on how well the quality criteria are fulfilled. The possible scores for the criteria of “inconsistency,” “indirectness,” “imprecision,” and “publication bias” are “undetected” (where all studies fulfilled the necessary criterion), “not serious” (where a majority of the studies fulfilled the criterion), “serious” (if less than half of the studies fulfilled the criterion), and “very serious” (where none of the studies fulfilled the required criterion).

The criterion of “risk of bias” consists of several subcriteria, which are scored differently to the other GRADE criteria listed previously. As the GRADE guidelines for assessment of risk of bias in studies was created to compare studies with an ideal RCT, a different set of subcriteria is necessary for nonrandomized studies. The risk of bias in Non-Randomised Studies of Interventions (ROBINS-I; Sterne et al. 2016) describes seven subcriteria which allow for the assessment of the risk of bias of nonrandomized controlled studies. These subcriteria are “bias due to confounding,” “selection bias,” “bias in measurement classification of interventions,” “bias due to deviation from intended interventions,” “bias due to missing data,” “bias in measurement of outcomes,” and “bias in selection of the reported result.” The assessment of the risk of bias of observational studies requires separate subcriteria as well, to judge the study against the correct standard. The GRADE guidelines offer 4 subcriteria which summarize the over 200 checklists and instruments which have been created to assess study limitations in observational studies. These subcriteria are “bias due to confounding,” “selection bias,” “bias due to measurement of exposure and/or outcome,” and “incomplete follow-up.” The possible scores for the risk of bias in subcriteria and individual studies are “low risk” (comparable with a well-designed RCT, i.e., low risk of bias for all domains), “moderate risk” (the study is sound for a nonrandomized study with regards to this domain, i.e., low or moderate risk of bias for all domains), “serious risk” (the study has some important problems, i.e., serious risk of bias in at least one domain, but not critical risk in any), and “critical risk” (the study is too problematic to provide any useful evidence on the effects of the intervention, i.e., critical risk of bias in at least one domain). A final risk of bias score is then determined for each outcome measure with the possible scores being: “undetected” (all criteria across studies are at a low risk of bias), “not serious” (most criteria across studies are at low risk of bias), “serious” (most criteria across studies are at moderate risk of bias), and “very serious” (most criteria across studies are at high risk of bias).

For each of the quality criteria for each measure, a judgment must be made as to whether the overall quality should be downgraded or, in rarer circumstances, upgraded. For each of the quality criteria, if no serious concerns exist, then no downgrading should take place. If serious concerns exist, then downgrade one level. If very serious concerns exist, then the quality should be downgraded two levels. It is possible to upgrade the quality rating if there is no more than a low risk for the study, and where there is a large magnitude of effect, or if the effect of all plausible confounding factors would be to reduce the effect size. A final overall score for the quality of evidence for each outcome measure is then given.

The GRADE guidelines also involve rating the importance of the outcomes with regard to the patient population in question and the outcomes’ importance for decision-making. This is predominantly for use by guideline developers; however, it is recommended of all researchers conducting systematic reviews (Guyatt et al. 2011a). The ratings can be “critical,” “important,” or “low importance.”

## RESULTS

**Fig. 1.**
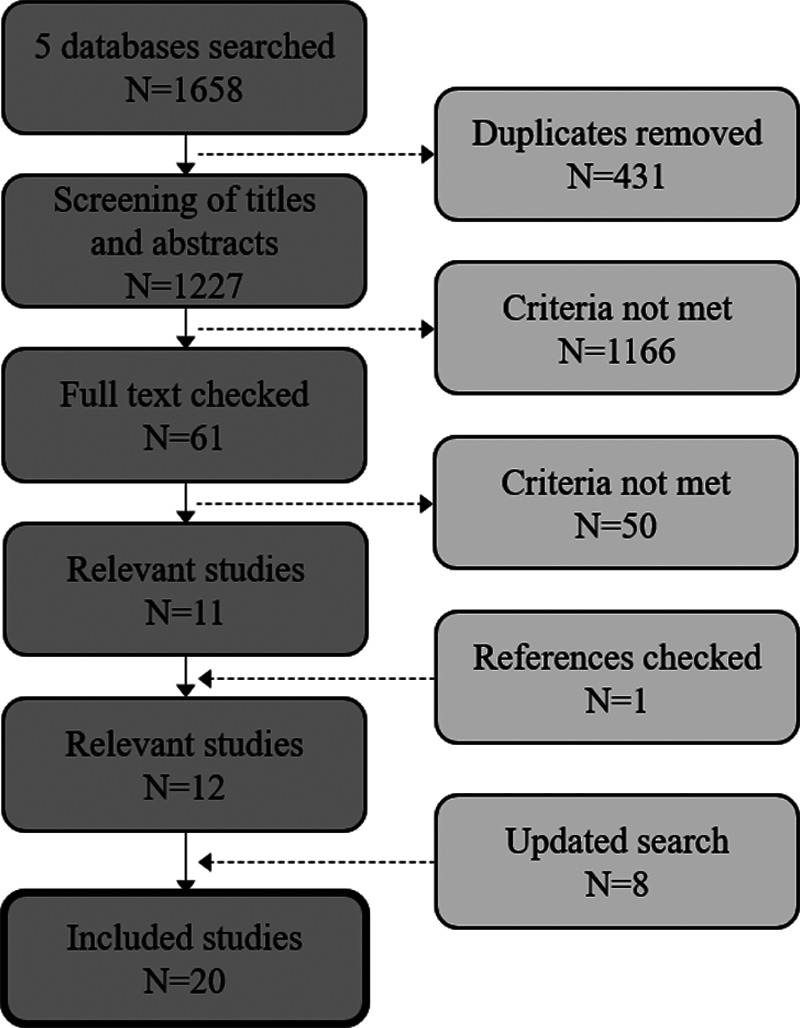
Identification of studies. The PRISMA flowchart of the process undertaken to select the relevant studies for this review is depicted.

The PRISMA flowchart in Figure 1 depicts the overall results from the search and the process involved in removing studies and selecting the final, relevant studies. The initial searches for both research questions produced 1,227 unique articles, after the removal of duplicates. The titles and abstracts of those articles were then screened, and a further 1,166 articles were removed. The majority of these 1,166 articles were removed because there was no measurement of fatigue (281), no measurement of hearing loss or hearing device use (259), or because the study specifically involved research into disease (176). The full texts of the remaining 61 studies were then examined, which resulted in 50 more articles being removed for not meeting the selection criteria. The reference lists of the remaining studies (11) were then examined to find any other studies that met the selection criteria. One was found using this method. Two updated searches were conducted, the final one on October 16, 2019, with eight further studies being selected. Thus a total of 20 studies were selected for this systematic review. One study provided evidence for both Q1 and Q2. The completed PRISMA checklist for systematic reviews can be found in Supplemental Digital Content 2, http://links.lww.com/EANDH/A677.

### General Characteristics

#### Population

In all of the studies, participants were people who were considered to have a hearing loss. With regards to the effect of hearing loss on fatigue (Q1), nine studies included both participants who had a hearing loss and participants who did not have a hearing loss (range n = 16 to 2,667, median n = 55). Three of these studies measured the level of hearing loss, rather than including those without a hearing loss as a separate group. Four studies included only participants with a hearing loss (range n = 84 to 3,216, median n = 1,528), and one study compared their data from participants with a hearing loss with normative data (n = 149). With regards to the effect of the hearing device fitting on fatigue, two studies measured participants with and without hearing devices (range n = 150 to 5,008, median n = 1,093). Four other studies measured the same group of participants twice, either before and after fitting, or as part of a crossover study (range n = 7 to 283, median n = 15.5). Three of these studies involved cochlear implants exclusively.

#### Interventions

The intervention of interest for Q1 was hearing loss. This was either the existence of hearing loss or the level of hearing loss. Varying thresholds were utilized to quantify hearing loss using either pure-tone audiometry or speech in noise tests. Self-reported hearing loss was used as the eligibility criterion by two studies. The intervention for Q2 was hearing device fitting. Six studies measured hearing device fitting against no hearing device fitting. One study compared the difference between using one and two hearing devices.

#### Comparators

The comparator for Q1 was either a group difference between participants with a hearing loss and those without, or within-group differences in the level of hearing loss. For Q2, the comparator was either the fitting of a hearing device versus no hearing device, or the difference between using one and two devices.

#### Outcomes

For the purposes of this review, transient (acute) fatigue was defined as fatigue experienced in a given moment, or immediately following the moment in question. Long-term fatigue was defined as either fatigue experienced over a period of time (e.g., the past week) or as average feelings of fatigue (e.g., how do you usually feel). The duration of fatigue measured for each study outcome type is displayed in Table 1.

**TABLE 1.** Number of findings from each type of outcome measure and type of fatigue measured

	Subjective Outcomes	Behavioral Outcomes	Physiological Outcomes
Long-term fatigue	30	0	0
Transient fatigue	1	1	0

##### Subjective

All subjective outcomes were self-report questionnaires, some of which were not validated. For the purposes of this review, questionnaires were considered validated if there was any published material demonstrating adequate reliability and validity of the measure in a representative sample (including healthy samples). Most of the questionnaires followed a Likert scale reporting system and consisted of between 1 and 10 responses. Six questionnaires consisted of dichotomous questions, offering a “yes” or “no” response. Three questionnaires measured fatigue using questions about energy, one questionnaire asked questions relating to anergia (a lack of energy), three questionnaires asked about vigor, and two questionnaires asked about “need for recovery.” The energy index of the “Nottingham Health Profile” (Wiklund 1990) was used in three studies, the “Fatigue Assessment Scale” (Michielsen et al. 2003) was used twice, the “Profile of Mood States” (McNair et al. 1971) was used twice, and the “Need for Recovery Scale” (van Veldhoven & Broersen 2003) was used twice. All other validated questionnaires were used only once. All studies, and the corresponding validated questionnaires used, are displayed in Supplemental Digital Content 3, http://links.lww.com/EANDH/A678.

##### Behavioral

One study included behavioral outcomes. Transient fatigue was measured based on performance in a dual-task paradigm, whereby a word recall task and a reaction time task were completed simultaneously. Slowing reaction times was described as signaling fatigue. In behavioral studies of fatigue, it is possible that fatigue-induced decrements in performance over time could be masked by learning effects in shorter trial blocks. However, the duration of the trial blocks in the study in question are adequately long to avoid this.

##### Physiological

No studies included physiological outcomes directly indicating fatigue. For example, Dwyer et al. (2019) and Wang et al. (2018), which were included in the review for their subjective outcomes, both utilized physiological measures. Dwyer et al. assessed salivary cortisol level, which is a marker of physiological stress. While this could lead to fatigue, it is not by itself a direct measure of fatigue. Likewise, Wang et al. used pupillometry to calculate peak pupil dilation (PPD). PPD measures effort and cognitive load. This again could potentially lead to fatigue, but PPD is not a direct measure of fatigue. However, changes in pupil dilation over time could be used to indicate fatigue. This technique has previously been used on adult (McGarrigle et al. 2017b) and child populations (McGarrigle et al. 2017a), however, only in normal-hearing participants.

Table 1 summarizes the number of findings associated with each type of outcome, measuring either long-term or transient fatigue.

#### Study Designs

This review included RCTs, non-RCTs, experimental studies with repeated measures design and observational studies. Qualitative studies were excluded. No RCTs were found. Thirteen observational studies were included. Three studies were prospective non-RCTs, two were repeated measures experiments, one was a diagnostic test study (a subset of observational design) and one was a crossover study (a subset of repeated measures design). Between-group test parameters were employed by thirteen studies; this was either hearing loss versus normal hearing, un-fitted versus fitted, or one versus two hearing devices. Nine studies utilized a within-group test parameter, which was always level of hearing loss. Two studies used both types of parameter.

### Results of Data Extraction and Management

For both Q1 and Q2, the methods of assessing fatigue were categorized as either subjective or behavioral methods. No physiological methods were found. The studies that fell into each category are listed alphabetically by first author in Table 2. In one study, both subjective and behavioral measures were used, so this study is repeated in the table. All other studies fell entirely into the subjective category. The subjective measures are further classified as coming from validated or nonvalidated questionnaires. The validated questionnaires are listed in Supplemental Digital Content 3, http://links.lww.com/EANDH/A678. Evidence pertaining to Q1 was provided by 24 findings from 15 studies. Evidence pertaining to Q2 was provided by 8 findings from 6 studies. One study provided evidence for both research questions. All outcomes were rated as “important” as they have an effect on the lives of people with a hearing loss. They were not listed as “critical” as the outcomes did not involve threat to life or serious medical impact (Guyatt et al. 2011a).

**TABLE 2.** Summary of included studies

Study	Study Design	Fatigue Measurement Response Scale (Range)—Number of Items—Validation	Test Parameters	H1 Findings (One Sign per Finding)	H2 Findings (One Sign per Finding)
Subjective measures					
Alhanbali et al. (2017)	OS	Likert (1–5) 10 – V	A	+	=, =
			C		
Alhanbali et al. (2018)	OS	Likert (1–5) 10 – V	B	=, +, +	
Bisgaard and Ruf (2017)	OS	Likert (1–5) 2 – NV	C		+
Cheng et al. (2008)	OS	Dichotomous (yes–no) 7 - NV	B	+	
Chung et al. (2012)	Non-RCT	Likert (1–6) 4 - V	C		+
Dalton et al. (2003)	OS	Likert (1–6) 4 - V	B	+, +	
Dwyer et al. (2019)	OS	Likert (0–4) 3 - NV	A	+, =	
		Likert (0–4) 7 - V			
Fredriksson et al. (2016)	D	Likert (1–5) 1 - NV	B	+	
Grimby and Ringdahl (2000)	OS	Dichotomous (yes–no) 3 - V	A	+	
Härkönen et al. (2015a)	Non-RCT	Likert (1–5) 1 - NV	D		+
Härkönen et al. (2015b)	Non-RCT	Likert (1–5) 1 - NV	C		+
Hornsby (2013)	C	Likert (0–10) 5 - NV	C		=
Hornsby and Kipp (2016)	OS	Likert (0–4) – V	A	=, +, =, +	
		2 validated questionnaires	B		
Jahncke and Halin (2012)	2x2 mixed factorial experiment	Likert (1–4) 3 – NV	A	+	
		Uses a validated measure in a NV way			
Karinen et al. (2001)	OS	Dichotomous (yes–no) 3 - V	A	+	
Nachtegaal et al. (2009)	OS	Dichotomous (yes–no) 11 - V	B	+	
Ringdahl and Grimby (2000)	OS	Dichotomous (yes–no) 3 - V	A	+	
Svinndal et al. (2018)	OS	Likert (0–3) 11 - V	B	+	
Wagner-Hartl and Kallus (2018)	RM	VAS (0–51) – NV (no published validation can be found)	B	=	
Wang et al. (2018)	OS*	Dichotomous (yes–no) 11 – V	A	=, =, =	
		Likert (1–7) 20 - V	B		
Behavioral measures					
Hornsby (2013)	C	Dual-task paradigm	C		+

A, HL vs. NH; B, level of HL; C, HD vs. 0HD; D, 1HD vs. 2HD; “+”, Hypothesis supported; “-”, Hypothesis refuted; “=”, no effect; H1: Hypothesis 1; H2: Hypothesis 2.

D, diagnostic test study; MF, mixed factorial experiment; NV, nonvalidated; *, observational (for the outcomes of interest); OS, observational study; RM, repeated measures experiment; VAS, visual analog scale; C, crossover; Non-RCT, prospective non-randomized controlled trial; V, validated.

For both of the research questions in this review, the evidence provided was from very different methodological approaches and inconsistent outcome measures. This lack of homogeneity meant that it was not possible to create reliable confidence intervals for each outcome. Therefore, following the approach adopted by Knudsen et al. (2010) and Ohlenforst et al. (2017), for each measurement type, the numbers of results that either support the hypothesis (+), show no effect (=) or show a negative effect (-) were simply summed and compared (Table 2, full output in Supplemental Digital Content 3, http://links.lww.com/EANDH/A678).

### Evidence on the Effect of Hearing Loss on Fatigue (Q1)

All evidence relating to the effect of hearing loss on fatigue came from subjective measures. The findings are summarized in Table 3. Of a total of 24 findings, 16 supported the hypothesis that hearing loss is associated with higher levels of fatigue and 8 findings were not statistically significant (i.e., did not support or refute the hypothesis). Eleven findings compared normal-hearing groups with hearing loss groups. Thirteen findings measured fatigue scores against the level of hearing loss. Nineteen findings came from validated questionnaires. Five findings came from nonvalidated questionnaires. None of the findings from self-report questionnaires indicated a reverse relationship (i.e., hearing loss being associated with reduced levels of fatigue).

**TABLE 3.** Characteristics of Q1 findings

	Findings Supporting H1	No Significant Effect	Findings Refuting H1
Total findings	16	8	0
HL group vs. NH group	7	4	0
Level of HL	9	4	0
Validated questionnaires	12	7	0
Non-validated questionnaires	4	1	0

H1, Hypothesis 1; HL, hearing loss; NH, normal hearing.

### Quality of Evidence on Q1

The GRADE evidence results for Q1 (the effect of hearing loss on fatigue) are given in Table 4. For each measurement type, the evidence was rated for five quality criteria (“risk of bias,” “inconsistency,” “indirectness,” “imprecision,” and “publication bias”) with four possible ratings (“undetected,” “not serious,” “serious,” and “very serious”). As the studies used so many different variations of self-report questionnaire, these are addressed together.

**TABLE 4.** Systematic review and GRADE results for Q1

Number of Studies	Risk of Bias	Inconsistency	Indirectness	Imprecision	Publication Bias	HL	NH	Quality	Importance
Quality of evidence for Q1									
Subjective measures									
11 (OS)	Not serious	Very serious	Serious	Serious	Undetected	6,344*	6,297*	Very low	Important
4 (Non-RCT)	Not serious								

*Includes populations where specific numbers of HL and NH participants are not given, and correlations are calculated. Those participants are therefore represented in both columns.

HD, hearing device wearer (number of participants in all studies); HL, hearing loss (number of participants in all studies); NH, normal hearing (number of participants in all studies); Non-RCT, nonrandomized controlled trial; OS, observational studies.

The criterion “risk of bias” consisted of subcriteria which were different depending on the design of the study. The risk of bias for observational studies was rated on “bias due to confounding,” “selection bias,” “bias due to measurement of exposure and/or outcome,” and “incomplete follow-up.” The risk of bias for nonrandomized controlled studies was rated on “bias due to confounding,” “selection bias,” “bias in measurement classification of interventions,” “bias due to deviation from intended interventions,” “bias due to missing data,” “bias in measurement of outcomes,” and “bias in selection of the reported result.” Eleven studies were at “moderate risk” or greater of “bias due to confounding,” most commonly due to a lack of controlling for hearing device use in either the study design or statistical analysis (Grimby & Ringdahl 2000; Ringdahl & Grimby 2000; Dalton et al. 2003; Cheng et al. 2008; Nachtegaal et al. 2009; Jahncke & Halin 2012; Fredriksson et al. 2016; Hornsby & Kipp 2016; Alhanbali et al. 2018; Svinndal et al. 2018; Wang et al. 2018). Most studies had a “low risk” of “selection bias.” Three studies had a “moderate” or “severe risk” due to a failure to match the population with the normative data used as the comparator (Grimby & Ringdahl 2000; Hornsby & Kipp 2016; Wagner-Hartl and Kallus 2018). Most of the additional criteria were at “low risk” of bias. The exceptions were a “moderate risk” of bias for four studies on the subcriterion “bias in measurement of exposure/outcomes” due to no hearing acuity data for the control group (Grimby & Ringdahl 2000; Ringdahl & Grimby 2000; Karinen et al. 2001; Hornsby & Kipp 2016) and for one study on the subcriterion “bias in selection of reported result” due to reporting an effect for only one item (Jahncke & Halin 2012). Overall, the risk of bias for all evidence on Q1 was rated as “not serious.”

The criterion “inconsistency” was rated as “very serious” due to large variations between studies on populations (e.g., normative data versus control groups), measurement of hearing loss (e.g., pure-tone average versus hearing handicap), measurements and terminology used for fatigue (e.g., fatigue, anergia, need for recovery, vitality), and study design. “Indirectness” was rated as “serious” due to selective populations being used (e.g., working adults, elderly) and surrogate measures of fatigue being used (e.g., vitality, need for recovery). The criterion “imprecision” was rated as “serious” as power sufficiency was rarely documented and the sample size was very small (16) for one study (Dwyer et al. 2019), while “publication bias” was “undetected.” As the evidence contains observational studies, it started as “low quality.” Given the limitations and risks of bias highlighted, the quality of evidence for Q1 was downgraded to “very low quality.”

### Evidence on the Effect of Hearing Device Fitting on Fatigue (Q2)

#### Subjective Measures

A majority of the findings relating to the effect of hearing device fitting on fatigue came from subjective measures (Table 5). Of a total of seven findings, four findings from subjective measures supported the hypothesis that hearing device fitting is associated with lower levels of fatigue, while three did not support the hypothesis. Four findings related to cochlear implant fitting and three related to hearing aid fitting. Two of the findings compared individuals’ fatigue scores pre and post fitting with a first cochlear implant. One finding compared individuals’ fatigue scores pre and post fitting with a second cochlear implant. Four findings compared groups with a hearing loss without a hearing device against groups with a hearing loss with a hearing device. Three findings came from validated questionnaires and four findings came from nonvalidated questionnaires.

**TABLE 5.** Characteristics of Q2 findings from subjective measures

	Findings Supporting H2	No Significant Effect	Findings Refuting H2
Subjective measures			
Total findings	4	3	0
Cochlear implants	3	1	0
Hearing aids	1	2	0
Pre & post 1st CI	2	0	0
Pre & post 2nd CI	1	0	0
HL aided vs. HL unaided	1	3	0
Validated questionnaires	1	2	0
Non-validated questionnaires	3	1	0

H2, Hypothesis 2; HL, hearing loss; CI, cochlear implant.

#### Behavioral Measures

Only one study produced a finding using behavioral measures, pertaining to Q2 (Hornsby 2013). The study used a dual-task paradigm and a crossover study design, involving a word recognition and recall task as well as a reaction time task. This finding supported the hypothesis that hearing device, in this case hearing aid fitting, is associated with decreased levels of fatigue.

### Quality of Evidence on Q2

The GRADE evidence results for Q2 are given in Table 6. Two measurement types were identified that address Q2. There were six studies that used subjective measures and one study that used behavioral measurement. As was done for Q1, for each measurement type the evidence was rated for five quality criteria (“risk of bias,” “inconsistency,” “indirectness,” “imprecision,” and “publication bias”) with four possible ratings (“undetected,” “not serious,” “serious,” and “very serious”). The quality of evidence for each measurement type was then assessed from these results.

**TABLE 6.** Systematic review and GRADE results for Q2

Number of Studies	Risk of Bias	Inconsistency	Indirectness	Imprecision	Publication Bias	HL	HD	Quality	Importance
Quality of evidence for Q2									
Subjective measures									
2 (OS)	Not serious	Serious	Serious	Serious	Undetected	2,456*	3,294*	Very low	Important
4 (Non-RCT)	Not serious								
Behavioral measures									
1 (Non-RCT)	Not serious	Undetected	Undetected	Serious	Undetected	16*	16*	Very low	Important

*Includes populations from crossover and prospective trials where participants fall into one group and then the other.

HD, hearing device wearer (number of participants in all studies); HL, hearing loss (number of participants in all studies); Non-RCT, nonrandomized controlled trial; OS, observational studies.

#### Quality of Evidence for Subjective Measures (Q2)

As the studies used so many different variations of self-report questionnaire, these are addressed together. The quality criterion “risk of bias” was judged separately for non-RCTs and observational studies, as in Q1. Both non-RCTs and observational studies were rated as “not serious.” Each subcriterion was rated as “low risk” other than “bias due to confounding” and “measurement of exposure and/or outcomes” which were rated as “moderate” due to no control for age (Härkönen et al. 2015a, 2015b; Bisgaard & Ruf 2017) and prior knowledge of intervention (Chung et al. 2012; Hornsby 2013; Härkönen et al. 2015a, 2015b). The criterion “inconsistency” was rated as “serious” due to different interventions and populations (e.g., 0, 1, or 2 hearing devices, as well as new or experienced users). “Indirectness” was rated as “serious” as surrogate fatigue measures were used (e.g., vitality), and selective populations were measured (e.g., working population). The criterion “imprecision” was rated as “serious” as power sufficiency was rarely provided. Publication bias was undetected. As the evidence contained observational studies, it started as “low quality.” Given the limitations and risks of bias highlighted, the quality of evidence for subjective measures investigating Q2 was downgraded to “very low” quality.

#### Quality of Evidence for Behavioral Measures (Q2)

Only one study was found to use behavioral measurement of fatigue to address Q2. The “risk of bias” was found to be “not serious” as only controlling for age and prior knowledge of the intervention were found to bias the respective subcriteria. The criteria of “inconsistency,” “indirectness,” and “publication bias” were rated as “undetected.” “Imprecision” was rated as “serious” due to a low sample size, and therefore low power. As the evidence comes from only one study, and “imprecision” is rated as “serious,” the quality of the evidence was downgraded to “very low” quality.

## DISCUSSION

This systematic review aimed to investigate the available evidence regarding two research questions: (Q1) Does hearing loss have an effect on fatigue? (Q2) Does hearing device fitting have an effect on fatigue? It was hypothesized that hearing loss results in increased fatigue (H1) and that hearing device fitting results in reduced fatigue (H2).

### Q1 Outcome Measures

#### Evidence and Quality of Evidence From Subjective Measures

Only studies utilizing subjective measures to investigate Q1 were found. As the search terms did not include the terms “performance” or “reduction in performance,” it is possible that relevant behavioral studies which did not use fatigue terminology could have been missed. All subjective measures assessed long-term fatigue, as opposed to transient fatigue. The evidence found from across these studies gives some support for H1; that hearing loss results in increased fatigue. Despite the small number of findings, the volume of positive results suggests that the research question is promising, particularly given the lack of any negative findings (hearing loss resulting in less fatigue). One of the possible reasons that more positive results were not found could be the large variation in the questionnaires used. Ten different questionnaires were used across the studies with the most commonly used questionnaire (the Nottingham Health Profile) being utilized by three studies. In addition, different forms of questionnaire were used (e.g., dichotomous questions versus visual analog scale), as well as varying ranges and varying terminologies. While superficially all the questionnaires might seem to be addressing the same topic, terminology such as loss of energy, need for recovery, and vitality each address slightly different aspects of fatigue. The “very low” GRADE rating for the quality of the evidence was mostly due to the lack of homogeneity among the studies and the fact that the systematic search found only non-RCTs (which are rated lower automatically). To reach a point where research outcomes can be reliably compared, more standardization is needed in terms of terminology and methodology. Equally, as more information is gathered regarding behavioral and physiological measurement of fatigue in normal-hearing populations, the use of these methodologies should be explored with regards to the impact of hearing loss on fatigue (Hopstaken et al. 2015; McGarrigle et al. 2017b; Herlambang et al. 2019).

### Q2 Outcome Measures

#### Evidence and Quality of Evidence From Subjective Measures

The majority of studies investigating Q2 utilized subjective measures. Almost all subjective measures assessed long-term fatigue; only one nonvalidated questionnaire measured transient fatigue. The evidence from the subjective measures did not support H2 in full; that hearing device fitting results in reduced fatigue, although the evidence is more promising for cochlear implant fitting than hearing aids. It must be noted that hearing device fitting does not necessarily equate to hearing device use, and any reduction of fatigue from the use of a hearing devices could be masked by those who do not use their devices regularly. An issue with the evidence from subjective measures pertaining to Q2 was the dearth of relevant literature. As the studies have the same lack of consistency in measuring fatigue as highlighted for Q1, as well as measuring different populations (hearing aid users versus cochlear implant users), results are even harder to compare and synthesis. The “very low” GRADE rating for the quality of the evidence is again mostly due to the lack of homogeneity in the studies, the small number of studies found and the fact the systematic search found only non-RCTs (which are rated lower automatically).

#### Evidence and Quality of Evidence From Behavioral Measures

Only one study used behavioral measures to investigate Q2. This study assessed transient fatigue, as opposed to long-term fatigue and supported H2. However, given that there is only one result from one study, no overall summary conclusions can be made. Likewise, ratings of the quality of the evidence are far less meaningful, given the identification of only one study that is not an RCT.

#### Summary of Evidence and Quality of Evidence on Q2

Overall the evidence on Q2 is sparse. The lack of evidence, and the low quality of this evidence, means that wider conclusions cannot be drawn. However, the positive result from the behavioral measure in particular means that further studies should be conducted to try and replicate the results using behavioral methodologies investigating transient fatigue. More consistency in measurement between studies is almost certainly required to obtain a clearer picture of the relationship between hearing device fitting and fatigue.

### Limitations of the Evidence

This review highlights the lack of existing information related to the relationship between hearing loss, hearing device fitting, and fatigue. Given that fatigue was not the main research aim of some studies, the measurement of fatigue was not always sufficiently thorough to support confidence in the comparison of conclusions. A serious limitation which was known before the search took place, and which the review has highlighted, is the difficulty measuring a construct as complex as fatigue. Studies used many different questionnaires to measure fatigue as well as using varied terminology. This could mean that some variables measured were not directly comparable. Instead these variables could be merely related, or part of a larger concept. More consistent terminology needs to be used regarding fatigue to allow solid conclusions to be drawn.

The majority of the studies in the review used self-report questionnaires to measure fatigue. All but one of the subjective findings measured long-term fatigue as opposed to transient fatigue. Hornsby (2013) used behavioral and subjective methodologies to investigate transient fatigue, the only behavioral methodology utilized in the review. More research using objective measures such as dual-task paradigms would put this result into better context.

### Strengths and Limitations of the Review

The search terms for the review included many different possible terms for fatigue as it was understood that studies used a wide variety. Given the lack of common understanding about fatigue as a concept and the potential semantic differences between terms, this almost certainly meant that several of the chosen studies did not measure the same thing. This was seen as unavoidable given the dearth of research available, but also as important to highlight the issue of defining fatigue in research. The inclusion criteria provided the possibility to include studies which measured performance decrement over time. However, only one such study was found, which was surprising given the recent increase in studies investigating listening effort using performance-based tasks. The search terms did not include the terms “performance” or “reduction in performance” as it was deemed that the large increase in search result size would not have significantly enhanced the search results. Thus, it is possible that some relevant studies involving a fatigue-related reduction in performance might have been missed due to not having used the included fatigue and performance reduction-related terms. Several other studies reported fatigue as a potential research outcome; however, only listening effort was measured. As there is no solid evidence of a direct link between listening effort and fatigue, these studies could not be included.

According to the GRADE protocol, the ratings of evidence provided in this review are “very low”: the worst rating. This is not a reflection on the individual studies themselves however, but stems from the fact that the majority of the studies were observational or non-RCTs. The GRADE system starts outcomes from this type of research at a rating of “low” quality. As fatigue was often measured as one of many health-related outcomes, the studies tended to be very different from one another in terms of population, measurement of fatigue, and measurement of hearing loss. This meant that it was almost inevitable that the quality of evidence would be downgraded.

The follow-up searches on March 1, 2019, and October 16, 2019, revealed eight new studies, which is a comparatively large proportion of the total number. This suggests that the volume of research in the area is growing, and thus this review is timely as a spur to the execution of more consistent studies in the future.

## CONCLUSIONS

One of the main conclusions of this systematic review is that the volume of evidence regarding the impact of hearing loss and hearing device fitting on fatigue is very small and the research area is in its early stages. This, combined with the diversity of studies and the “very low” quality of evidence generated, means that solid conclusions are not possible at this stage. There is some support given to H1 (hearing loss results in increased fatigue) and H2 (hearing device fitting results in reduced fatigue) however as a majority of results were positive and no results were negative, albeit nonsignificant. The majority of studies used subjective measures to investigate fatigue, with only one study using behavioral measures (Hornsby 2013) and none using physiological measures. As subjective and objective behavioral measures of fatigue did not appear to correlate, it is possible that different aspects of fatigue are being measured by each measurement type. More research is needed into what is being measured by subjective and objective assessment of fatigue, including the suitability for measuring long-term and transient fatigue. Given the increasing number of publications successfully investigating fatigue in normal-hearing populations using behavioral and physiological measures, these methodologies should be used more in the future to investigate fatigue in populations with a hearing loss. This review highlights that the weight of existing knowledge exists in the subjective measurement of long-term fatigue. As such, this is an area where future research should look to use validated measures and consistent terminology to build upon the comparatively larger evidence.

## ACKNOWLEDGMENTS

This work was supported by the Medical Research Council [grant numbers MR/R502169/1, MR/S003576/1]; and by the Chief Scientist Office of the Scottish Government. The funder had no role in study design.

## Supplementary Material


