# Supplementary material for: The Effect of Hearing Loss and Hearing Device Fitting on Fatigue in Adults: A Systematic Review
Source: Ear Hear. 2020 Jul 2;42(1):1–11. doi: 10.1097/AUD.0000000000000909 (PMC7757744; doi:10.1097/AUD.0000000000000909)
Supplement: Supplementary file 1 [file aud-42-001-s001.docx]

**Supplemental Digital Content 1. Literature search full terms (in output form)**

**EMBASE Fatigue Literature search**

| 1. exp fatigue/co, di, dm, ep, et, pc, rh, si, th [Complication, Congenital Disorder, Diagnosis, Disease Management, Epidemiology, Etiology, Prevention, Rehabilitation, Side Effect, Therapy] | | | |  |
| --- | --- | --- | --- | --- |
| 2. (tired* or vigo?r or exhaust* or letharg*).mp. [mp=title, abstract, heading word, drug trade name, original title, device manufacturer, drug manufacturer, device trade name, keyword, floating subheading] | | | |  |
| 3. (energy adj3 (lack or reduc* or less or loss or decrease* or lower* or mental or cognitive)).mp. [mp=title, abstract, heading word, drug trade name, original title, device manufacturer, drug manufacturer, device trade name, keyword, floating subheading] | | | |  |
| 4. ("listening effort" or "perceptual effort" or "ease of listening" or "cognitive effort" or "cognitive load").mp. [mp=title, abstract, heading word, drug trade name, original title, device manufacturer, drug manufacturer, device trade name, keyword, floating subheading] | | | |  |
| 5. exp hearing impairment/co, cn, di, dm, ep, et, pc, rh, si, th [Complication, Congenital Disorder, Diagnosis, Disease Management, Epidemiology, Etiology, Prevention, Rehabilitation, Side Effect, Therapy] | | | |  |
| 6. deaf*.mp. | | | |  |
| 7. (hearing adj3 (loss or impair*)).mp. [mp=title, abstract, heading word, drug trade name, original title, device manufacturer, drug manufacturer, device trade name, keyword, floating subheading] | | | |  |
| 8. exp hearing aid/am, ct, dc, de [Adverse Device Effect, Clinical Trial, Device Comparison, Device Economics] | | | |  |
| 9. ("hearing aid*" or "hearing-aid*").mp. [mp=title, abstract, heading word, drug trade name, original title, device manufacturer, drug manufacturer, device trade name, keyword, floating subheading] | | | |  |
| 10. ("hearing device" or "hearing system" or "hearing instrument" or "cochlear implant*" or "otosclerosis surgery").mp. [mp=title, abstract, heading word, drug trade name, original title, device manufacturer, drug manufacturer, device trade name, keyword, floating subheading] | | | |  |
| 11. (hearing adj3 (loss or impair*) adj6 (amplif* or aided or unaided)).mp. [mp=title, abstract, heading word, drug trade name, original title, device manufacturer, drug manufacturer, device trade name, keyword, floating subheading] | | | |  |
|  | | |  |  |
|  | | |  |  |
|  | | |  |  |
|  | | |  |  |
| 12. 1 or 2 or 3 or 4 | | | |  |
| 13. 5 or 6 or 7 | | | |  |
| 14. 8 or 9 or 10 or 11 | | | |  |
|  | | | |  |
| 15. 12 and 13 (Fatigue and Hearing Impairment) | | | |  |
| 16. 12 and 14 (Fatigue and Hearing Aids) | | | |  |
|  | |  |  |  |
|  | |  |  |  |
|  | |  |  |  |
| 17. limit 15 to (human and english language and article and (adult <18 to 64 years> or aged <65+ years>)) | | | |  |
| 18. limit 16 to (human and english language and article and (adult <18 to 64 years> or aged <65+ years>)) | | | |  |
|  |  |  |  |  |
|  |  |  |  |  |
|  |  |  |  |  |

**MEDLINE Fatigue Literature Search**

| 1. exp Auditory Fatigue/ or exp Fatigue/ | | | |  |
| --- | --- | --- | --- | --- |
| 2. (tired* or vigo?r or exhaust* or letharg*).mp. [mp=title, abstract, original title, name of substance word, subject heading word, keyword heading word, protocol supplementary concept word, rare disease supplementary concept word, unique identifier] | | | |  |
| 3. (energy adj3 (lack or reduc* or less or loss or decrease* or lower* or mental or cognitive)).mp. [mp=title, abstract, original title, name of substance word, subject heading word, keyword heading word, protocol supplementary concept word, rare disease supplementary concept word, unique identifier] | | | |  |
| 4. ("listening effort" or "perceptual effort" or "ease of listening" or "cognitive effort" or "cognitive load").mp. [mp=title, abstract, original title, name of substance word, subject heading word, keyword heading word, protocol supplementary concept word, rare disease supplementary concept word, unique identifier] | | | |  |
| 5. exp Hearing Loss/cl, co, cn, di, ec, ep, eh, et, ge, nu, pa, pp, pc, px, rh, su, th, us [Classification, Complications, Congenital, Diagnosis, Economics, Epidemiology, Ethnology, Etiology, Genetics, Nursing, Pathology, Physiopathology, Prevention & Control, Psychology, Rehabilitation, Surgery, Therapy, Ultrasonography] | | | |  |
| 6. deaf*.mp. | | | |  |
| 7. (hearing adj3 (loss or impair*)).mp. [mp=title, abstract, original title, name of substance word, subject heading word, keyword heading word, protocol supplementary concept word, rare disease supplementary concept word, unique identifier] | | | |  |
| 8. exp Hearing Aids/ae, cl, ct, ec, es, is, mt, nu, px, st, sn, sd, td, ut [Adverse Effects, Classification, Contraindications, Economics, Ethics, Instrumentation, Methods, Nursing, Psychology, Standards, Statistics & Numerical Data, Supply & Distribution, Trends, Utilization] | | | |  |
| 9. ("hearing aid*" or "hearing-aid*").mp. [mp=title, abstract, original title, name of substance word, subject heading word, keyword heading word, protocol supplementary concept word, rare disease supplementary concept word, unique identifier] | | | |  |
| 10. ("hearing device" or "hearing system" or "hearing instrument" or "cochlear implant*" or "otosclerosis surgery").mp. [mp=title, abstract, original title, name of substance word, subject heading word, keyword heading word, protocol supplementary concept word, rare disease supplementary concept word, unique identifier] | | | |  |
| 11. (hearing adj3 (loss or impair*) adj6 (amplif* or aided or unaided)).mp. [mp=title, abstract, original title, name of substance word, subject heading word, keyword heading word, protocol supplementary concept word, rare disease supplementary concept word, unique identifier] | | | |  |
|  | | |  |  |
|  | | |  |  |
|  | | |  |  |
|  | | |  |  |
| 12. 1 or 2 or 3 or 4 | | | |  |
| 13. 5 or 6 or 7 | | | |  |
| 14. 8 or 9 or 10 or 11 | | | |  |
|  | | | |  |
| 20. 12 and 13 (Fatigue and Hearing Impairment) | | | |  |
| 21. 12 and 14 (Fatigue and Hearing Aids) | | | |  |
|  | |  |  |  |
|  | |  |  |  |
|  | |  |  |  |
| 20. limit 20 to (english language and humans and "all adult (19 plus years)" and journal article) | | | |  |
| 21. limit 21 to (english language and humans and "all adult (19 plus years)" and journal article) | | | |  |
|  |  |  |  |  |
|  |  |  |  |  |
|  |  |  |  |  |

**Web of Science Fatigue Literature Search**

| #16 | (#14) *AND* **LANGUAGE:** (English) *AND* **DOCUMENT TYPES:** (Article)  *DocType=All document types; Language=All languages;* |
| --- | --- |
| #15 | (#13) *AND* **LANGUAGE:** (English) *AND* **DOCUMENT TYPES:** (Article)  *DocType=All document types; Language=All languages;* |
| #14 | #12 AND #10  *DocType=All document types; Language=All languages;* (Fatigue and Hearing Aids) |
| #13 | #11 AND #10  *DocType=All document types; Language=All languages;* (Fatigue and Hearing Impairment) |
| #12 | #7 OR #6  *DocType=All document types; Language=All languages;* |
| #11 | #5 OR #4  *DocType=All document types; Language=All languages;* |
| #10 | #3 OR #2 OR #1  *DocType=All document types; Language=All languages;* |
| #7 | TS= (hearing NEAR (loss or impair*) NEAR (amplif* or aided or unaided))  *DocType=All document types; Language=All languages;* |
| #6 | TS= ("hearing aid*" or "hearing-aid*" or "hearing device" or "hearing system" or "hearing instrument" or "cochlear implant*" or "otosclerosis surgery")  *DocType=All document types; Language=All languages;* |
| #5 | ts=(hearing NEAR (loss OR impair*))  *DocType=All document types; Language=All languages;* |
| #4 | ts=(deaf*)  *DocType=All document types; Language=All languages;* |
| #3 | ts= ("listening effort" or "perceptual effort" or "ease of listening" or "cognitive effort" or "cognitive load")  *DocType=All document types; Language=All languages;* |
| #2 | ts= (energy NEAR (lack or reduc* or less or loss or decrease* or lower* or mental or cognitive))  *DocType=All document types; Language=All languages;* |
| #1 | ts=(fatigue OR tired* or vigo?r or exhaust* or letharg*)  *DocType=All document types; Language=All languages;* |

**PSYCINFO Fatigue Literature Search**

| S15 | S11 AND S13 | Limiters - English; Age Groups: Adulthood (18 yrs & older); Population Group: Human; Document Type: Journal Article  Search modes - Boolean/Phrase |
| --- | --- | --- |
| S14 | S11 AND S12 | Limiters - English; Language: English; Age Groups: Adulthood (18 yrs & older); Population Group: Human; Document Type: Journal Article  Search modes - Boolean/Phrase |
| S13 | S8 OR S9 OR S10 | Search modes - Boolean/Phrase |
| S12 | S5 OR S6 OR S7 | Search modes - Boolean/Phrase |
| S11 | S1 OR S2 OR S3 OR S4 | Search modes - Boolean/Phrase |
| S10 | hearing N3 (loss or impair*) N6 (amplif* or aided or unaided) | Search modes - Boolean/Phrase |
| S9 | "hearing aid*" or "hearing-aid*" or "hearing device" or "hearing system" or "hearing instrument" or "cochlear implant*" or "otosclerosis surgery" | Search modes - Boolean/Phrase |
| S8 | DE "Hearing Aids" OR DE "Cochlear Implants" | Search modes - Boolean/Phrase |
| S7 | deaf* | Search modes - Boolean/Phrase |
| S6 | hearing N3 (loss or impair*) | Search modes - Boolean/Phrase |
| S5 | DE "Deaf" | Search modes - Boolean/Phrase |
| S4 | "listening effort" or "perceptual effort" or "ease of listening" or "cognitive effort" or "cognitive load" | Search modes - Boolean/Phrase |
| S3 | (energy N3 (lack or reduc* or less or loss or decrease* or lower* or mental or cognitive)) | Search modes - Boolean/Phrase |
| S2 | tired* or vigo?r or exhaust* or letharg* | Search modes - Boolean/Phrase |
| S1 | DE "Fatigue" | Search modes - Boolean/Phrase |

**Cochrane Library Literature Search**

1. "Fatigue" OR "Energy" OR "listening effort"
2. "hearing loss"OR "hearing-loss" OR "hearing impairment" OR "hearing aids" OR "hearing-aids" OR "hearing device" OR "cochlear implant"
3. 1 AND 2
