# Supplementary material for: The Effect of Hearing Loss and Hearing Device Fitting on Fatigue in Adults: A Systematic Review
Source: Ear Hear. 2020 Jul 2;42(1):1–11. doi: 10.1097/AUD.0000000000000909 (PMC7757744; doi:10.1097/AUD.0000000000000909)
Supplement: Supplementary file 3 [file aud-42-001-s003.docx]

Supplemental Digital Content 3: Full systematic review results table

|  | **Study** | **num** | **Number of subjects** |  | **Mean age (years)** |  | **Type of hearing loss & measurement technique** |  | **Study Design** |  | **Supplementary measures**  **For fatigue: F**  **For Hearing: HL** |  | **Fatigue Measurement**  Type (range) number of items |  | **Test parameters**  A: HL vs. NH  B: Level of HL  C: HA vs 0HA  D: 1HA vs 2HA |  | **(H1) Fatigue: HL > NH**  **+: HP supported**  **-: HP not supported**  **=: no effect** |  | **(H2) Fatigue: aided < unaided**  **+: HP supported**  **-: HP not supported**  **=: no effect** |
| --- | --- | --- | --- | --- | --- | --- | --- | --- | --- | --- | --- | --- | --- | --- | --- | --- | --- | --- | --- |
|  | **Subjective measures** |  |  |  |  |  |  |  |  |  |  |  |  |  |  |  |  |  |  |
|  | Alhanbali et al (2016) |  | a) 50 SSD  b) 50 HA  c) 50 CI  d) 50 NH |  | Medians  a)68  b)72  c)71  d)71 |  | a)profound unilateral HL (<35dBHL better ear)  b)mild – severe SNHL  c)CI for +6 months  d)<30dBHL  (PTA) |  | Observational |  |  |  | Likert (1-5) 10 – V  ‘Fatigue Assessment Scale’ |  | **(A)**  **(C):** Both HA and CI vs HI |  | **1(+)** Significant group mean difference |  | **2(=, =)** No significant difference between CI, HA and SSD |
| 1. 2 | Alhanbali et al (2017) |  | n=84 HL |  | 72 |  | Mild to severe bilateral SNHL  (PTA) |  | Observational |  | **HI**: Hearing Handicap  Speech recognition in noise |  | Likert (1-5) 10 – V  ‘Fatigue Assessment Scale’ |  | **(B):** Fatigue correlated against PTA, Hearing Handicap & speech recognition in noise |  | **1(=)** No sig correlation fatigue with PTA  **1(+)** Sig correlation fatigue with handicap  **1(+)** Sig correlation fatigue with speech recognition |  |  |
| 1. 3 | Bisgaard (2017) |  | a)2085 HL  b)2923 HA |  | unknown |  | Self-reported Hearing Impairment  Upper 50% HL in HI group |  | Observational |  |  |  | Likert (1-5) 2 - NV |  | **(C)** |  |  |  | **2(+)** Significant differences between groups for each response other than “strongly disagree” |
|  | Cheng et al (2008) |  | n=2130 |  | 74 |  | Any – derived from medical history |  | Observational |  | **F:** Anaergia questions |  | Dichotomous (yes-no) 7 - NV |  | **(B)** |  | **1(+)** Significant odds ratio |  |  |
|  | Chung et al (2012) |  | n=283 |  | 52.88 |  | Not given. CI candidates (severe) |  | Prospective non-RCT |  | **F:** Vitality component of questionnaire |  | Likert (1-6) 6 – V  ‘36 item short form survey instrument’, Energy/fatigue component |  | **(C):** Measurements pre and post CI implantation |  |  |  | **2(+)** Significant group difference for all participants, and each age group other than elderly. |
|  | Dalton et al (2003) |  | n=2502 |  | 69 |  | Any including none  (PTA) |  | Observational |  | **F:** Vitality  **HI**: Hearing Handicap |  | Likert (1-6) 4 – V  ‘36 item short form survey instrument’, Energy/fatigue component |  | **(B)** Vitality correlated against Hearing handicap and PTA |  | **1(+, +)** Significant correlations of less vitality with PTA and Hearing Handicap |  |  |
|  | Dwyer et al (2019) |  | a) 8 HL  b) 8 NH |  | 24.3 |  | Severe – profound (PTA) |  | Observational |  |  |  | Likert (0-4) 3 - NV  Likert (0-4) 7 – V  ‘Profile of mood states’ |  | **(A)** |  | **1(+, =)** Significant group difference for listening fatigue, but not for general fatigue |  |  |
|  | Fredriksson et al (2016) |  | a) 26 HL  b) 29 NH |  | a)49  b)45 |  | >40dBHL at one frequency in either ear.  HINT SNR >-3dB  DPOAE <3dB |  | Diagnostic test study |  | **F:** Sound induced auditory fatigue |  | Likert (1-5) 1 – NV |  | **(B)** A diagnostic test of variables (auditory fatigue) against gold standard HL measurements |  | **1(+)** Highest sensitivity and specificity for auditory fatigue against PTA and DPOAE |  |  |
|  | Grimby et al (2000) |  | a)35 HL  b)1256 NH |  | a)42 |  | Severe-profound (PTA) |  | Observational |  | **F:** lack of energy |  | Dichotomous (yes-no) 3 – V  ‘Nottingham Health Profile’, energy level component |  | **(A)** |  | **1(+)** Significant group difference |  |  |
|  | Harkonen et al (2015) b |  | 7 |  | 48 |  | SSD CI patients  <20dBHL good ear  (PTA) |  | Prospective non-RCT |  |  |  | Likert (1-5) 1 - NV |  | **(C)** Pre and post unilateral cochlear implantation |  |  |  | **2(+)** Mode answer of a little improved fatigue |
|  | Harkonen et al (2015) a |  | 15 |  | 41 |  | Second CI patients |  | Prospective non-RCT |  |  |  | Likert (1-5) 1 - NV |  | **(D)** Pre and post second cochlear implantation |  |  |  | **2(+)** Mode answer of moderately improved fatigue |
|  | Hornsby (2013) |  | 16 |  | 65.8 |  | Mild-severe bilateral symmetric sloping SNHL  (PTA) |  | Crossover |  |  |  | Likert (0-10) 5 - NV |  | **(C)** |  |  |  | **2(=)** No significant group difference for pre-post fatigue scores |
|  | Hornsby et al (2016) |  | 149 (116 for elderly sub analysis) |  | 66 (72.3 for elderly sub analysis) |  | Any  (PTA) |  | Observational |  | **F:** Vitality  **HI:** Hearing Handicap |  | Likert (0-4) – V  2 validated questionnaires  ‘Profile of mood states’,  ‘Multidimensional fatigue symptom inventory – short form’ |  | **(A)**Test group vs Normative data  **(B)**Correlations against PTA and Hearing Handicap |  | **1(=, +, =, +)** Sig group effect for vitality not fatigue. Fatigue correlates (non-linear) with HH, but not PTA |  |  |
|  | Jahncke et al (2012) |  | a)20 HL  b)18 NH |  | a)53  b)48 |  | 4FAHL >28dBHL  (PTA) |  | 2x2 mixed factorial experiment |  |  |  | Likert (1-4) 3 – NV  Uses ‘Swedish occupational fatigue inventory’ in a NV way |  | **(A)** |  | **1(+)** 1 item “amount of yawning” sig higher for HI group in high noise condition |  |  |
|  | Karinen et al (2001) |  | 34 |  | Unknown  Range 24-74 |  | CI candidates  No measurements taken |  | Observational |  | **F:** energy |  | Dichotomous (yes-no) 3 – V  ‘Nottingham Health Profile’, energy level component |  | **(A)**HA group vs NH normative data |  | **1(+)** Visually the HA group scores worse than norms for energy |  |  |
|  | Nachtegaal et al (2009) |  | 925 |  | 44 |  | Good = SRT<5.5dB  Insufficient = SRT between -5.5 and -2.8 dB  Poor = SRT>-2.8dB  Internet based speech in noise test |  | Observational |  | **F:**Need for Recovery |  | Dichotomous (yes-no) 11 – V  ‘Need for recovery scale’ |  | **(B)** correlation between SRT and need for recovery |  | **1(+)** linear regression shows a positive correlation |  |  |
|  | Ringdahl et al (2000) |  | a)311 HL  b)2356 NH |  | a)66  b)age matched controls |  | Severe-profound Hearing loss  >70dBHL at 1kHz  (PTA) |  | Observational |  | **F:** energy |  | Likert (yes-no) 3 – V  ‘Nottingham Health Profile’, energy level component |  | **(A)** |  | **1(+)** Significant group difference |  |  |
|  | Svinndal et al (2018) |  | 3216 |  | 54.7 |  | Self-reported hearing loss (WHO classifications) |  | Observational |  |  |  | Likert (1-4) 11 – V  ‘Chalder’s fatigue scale’ |  | **(B)** |  | **1(+)** Logistic regression shows increased likelihood of higher fatigue with higher HL |  |  |
|  | Wagner-Hartl et al (2017) |  | 51 |  | 40 |  | ≥15 dBHL in 2 or more of 0.5, 1, 2, 3, 4kHz  (PTA) |  | Repeated measures experiment |  |  |  | VAS (0-51) – NV (no published validation can be found) |  | **(B)** correlation between fatigue and PTA |  | **1(=)** No significant relationship |  |  |
|  | Wang et al (2018) |  | a)19 HL  b) 27 NH |  | a)47.2  b)46.3 |  | Sensorineural symmetrical HL between 35 and 65 dBHL 4FAHL  NH ≤ 20dBHL 5FAHL  (PTA & SRT) |  | Observational (for the outcomes of interest) |  | **F:** Need for Recovery |  | Dichotomous (yes-no) 11 – V  Likert (1-7) 20 – V  ‘Need for recovery’,  ‘Checklist individual strength’ |  | **(A)**HI vs NH for 2 questionnaires  **(B)**Correlation between level of HL/SRT and questionnaires |  | **1(=, =, =)**  No significant group difference or correlation (PTA or SRT) |  |  |
|  | ***Objective measures*** |  |  |  |  |  |  |  |  |  |  |  |  |  |  |  |  |  |  |
|  | Hornsby (2013) |  | 16 |  | 65.8 |  | Mild-severe bilateral symmetric sloping SNHL  (PTA) |  | Crossover |  | **F:** Increasing reaction times |  | Dual Task Paradigm: Word recognition, Word Recall and visual reaction times |  | **(C)** |  |  |  | **2(+)** Significant increase in reaction times (fatigue) over time in unaided group and not in aided group |

HL: Hearing loss; NH: Normal hearing; CI: Cochlear Implant; SSD: Single sided deafness; PTA: pure tone audiometry; SRT: Speech reception threshold; SNHL: Sensorineural hearing loss; V: Validated; NV: Non-Validated; A: HL vs. NH; B: Level of HL; C: HA vs 0HA; D: 1HA vs 2HA; “+”: Hypothesis supported; “-”: Hypothesis refuted; “=”: no effect; H1: Hypothesis 1; H2: Hypothesis 2
